# *CYP2B6* Genotype and Weight Gain Differences Between Dolutegravir and Efavirenz

**DOI:** 10.1093/cid/ciaa1073

**Published:** 2020-09-22

**Authors:** Rulan Griesel, Gary Maartens, Maxwell Chirehwa, Simiso Sokhela, Godspower Akpomiemie, Michelle Moorhouse, Francois Venter, Phumla Sinxadi

**Affiliations:** 1 Division of Clinical Pharmacology, Department of Medicine, University of Cape Town, Cape Town, South Africa; 2 Wellcome Centre for Infectious Diseases Research in Africa, Institute of Infectious Disease and Molecular Medicine, University of Cape Town, Cape Town, South Africa; 3 Ezintsha, Wits Reproductive Health and HIV Institute, Faculty of Health Sciences, University of the Witwatersrand, Johannesburg, South Africa

**Keywords:** efavirenz, dolutegravir, CYP2B6, weight, dual-energy x-ray absorptiometry

## Abstract

**Background:**

Dolutegravir is associated with more weight gain than efavirenz. Loss-of-function polymorphisms in *CYP2B6* result in higher efavirenz concentrations, which we hypothesized would impair weight gain among people living with human immunodeficiency virus (HIV; PLWH) starting efavirenz-based antiretroviral therapy (ART).

**Methods:**

We studied ART-naive participants from the ADVANCE study randomized to the efavirenz /emtricitabine/tenofovir disoproxil fumarate (TDF) and dolutegravir/emtricitabine/TDF arms. We compared changes in weight and regional fat on DXA from baseline to week 48 between *CYP2B6* metabolizer genotypes in the efavirenz arm, and with the dolutegravir arm.

**Results:**

There were 342 participants in the dolutegravir arm and 168 in the efavirenz arm who consented to genotyping. Baseline characteristics were similar. Weight gain was greater in women than men. In the efavirenz arm *CYP2B6* metaboliser genotype was associated with weight gain (*P* = .009), with extensive metabolizers gaining the most weight, and with changes in regional fat in women, but not in men. Weight gain was similar in *CYP2B6* extensive metabolizers in the efavirenz arm and in the dolutegravir arm (*P* = .836). The following variables were independently associated with weight gain in all participants: baseline CD4 count, baseline human immunodeficiency virus type 1 (HIV-1) RNA, and *CYP2B6* metaboliser genotype.

**Conclusions:**

*CYP2B6* metaboliser genotype was associated with weight gain in PLWH starting efavirenz-based ART. Weight gain was similar between *CYP2B6* extensive metabolizers in the efavirenz arm and in the dolutegravir arm, suggesting that impaired weight gain among *CYP2B6* slow or intermediate metabolizers could explain the increased weight gain on dolutegravir compared with efavirenz observed in ADVANCE and other studies.

## Teaser

*CYP2B6* metabolizer genotype was associated with weight gain and changes in regional fat in women, but not in men starting efavirenz-based antiretroviral therapy (ART). Weight gain was similar in *CYP2B6* extensive metabolizers in the efavirenz arm and in the dolutegravir arm.

**(See the Editorial Commentary by Conway on pages e3910–1.)**

Integrase strand transfer inhibitors (InSTIs) are associated with more weight gain than other classes of antiretrovirals in people living with human immunodeficiency virus (HIV; PLWH) starting antiretroviral therapy (ART) [1–3]. Dolutegravir, a second generation InSTI, is associated with more weight gain than the first generation InSTIs [1–4]. Dolutegravir is replacing efavirenz in first-line ART in resource-limited settings and second generation InSTIs are also widely used in high-income countries. Female sex and Black race are risk factors for more weight gain in PLWH starting ART [1, 4] and switching [5] to InSTI-based ART. Two randomised controlled trials of dolutegravir conducted in sub-Saharan Africa recently reported their 48-week findings, including weight gain. The NAMSAL study, conducted in Cameroon, randomised ART-naive participants to dolutegravir or efavirenz at the low dose of 400 mg, both combined with tenofovir disoproxil fumarate (TDF) and lamivudine, and reported greater increases in body weight and treatment-emergent obesity in the dolutegravir arm [6]. The ADVANCE study conducted in South Africa, randomized ART-naive participants to dolutegravir or efavirenz at the standard dose of 600 mg, both combined with emtricitabine and TDF, and a third arm of dolutegravir combined with emtricitabine and tenofovir alafenamide (TAF): increases in body weight were greater in the dolutegravir arms, especially in the dolutegravir/emtricitabine/TAF arm [7]. The magnitude of the weight gain over the short term in the dolutegravir arms in ADVANCE and NAMSAL, especially in female participants, has caused concern as obesity is a major risk factor for multiple noncommunicable diseases, which disproportionately affect sub-Saharan Africa [8].

The greater weight gain observed with dolutegravir than efavirenz could be due to off target metabolic effects of dolutegravir or to impaired weight gain in PLWH on efavirenz. Efavirenz causes several metabolic toxicities [9], including concentration dependent mitochondrial toxicity and impaired adipocyte differentiation [10], which could impair weight gain. Efavirenz was associated with less limb fat gain than protease inhibitors in a systematic review of randomized controlled trials [11]. Chronic neuropsychiatric efavirenz toxicity could impair appetite. Efavirenz is primarily metabolized by the cytochrome P450 2B6 enzyme (CYP2B6). Three genetic loss-of-function polymorphisms in *CYP2B6* have been identified that result in higher efavirenz concentrations [12]. A cohort study reported that PLWH who had *CYP2B6* slow metabolizer genotypes gained more weight when switched from efavirenz- to InSTI-based ART [13], suggesting that high efavirenz exposure could impair weight gain.

We hypothesized that *CYP2B6* metabolizer genotypes associated with higher efavirenz concentrations would impair weight gain in PLWH starting efavirenz-based ART. We determined the effect of *CYP2B6* metabolizer genotype on weight gain in a subset of participants randomised to efavirenz in the ADVANCE study and compared their weight gain, categorised by *CYP2B6* metabolizer genotype, with participants randomized to the dolutegravir arm with the same nucleoside reverse transcriptase inhibitors (emtricitabine and TDF).

## METHODS

### Study Design and Participants

ADVANCE was an open-label, 3 arm randomized trial performed in Johannesburg, South Africa [7], which compared dolutegravir and emtricitabine plus either TDF or TAF with efavirenz and emtricitabine plus TDF. Enrolled participants were ≥12 years, had not received ART in the previous 6 months, had a creatinine clearance of >60 mL/minute, and human immunodeficiency virus type 1 (HIV-1) RNA ≥ 500 copies/mL.

Participants in the efavirenz arm who consented to genetic testing and participants randomized to the dolutegravir arm with emtricitabine plus TDF were included in this substudy. Weight measurements obtained at week 4, 12, 24, 36, and 48 were used to calculate the percentage change in weight from baseline. Pregnancy during the 48-week observation period was an exclusion criterion. Body composition measures using dual-energy X-ray absorptiometry (DXA) (Discovery DXA System®, software version APEX 4.6.0.1, Hologic, Bedford, MA, USA) at baseline and week 48 were used to estimate changes in fat and lean body mass. DXA cutoff lines positioned at anatomical markers were used to obtain fat mass for the whole body as well as for regions of interest (arms, legs, and trunk). The trunk comprised the region between the inferior edge of the chin and the middle of the femoral necks (without touching the brim of the pelvis), excluding the arms. Limb composition was determined by summing the arms and legs. Abdominal visceral adipose tissue (VAT) and subcutaneous adipose tissue (SAT) were estimated by a single trained analyst using a validated method [14]. The percentage change in mass from baseline to week 48 was calculated for limb and trunk fat, VAT and SAT, and lean body mass.

### Determination and Characterization of Genetic Polymorphisms

DNA extraction from whole blood samples was done by the salting out method. Genotyping was done by MassARRAY® single nucleotide polymorphism (SNP) genotyping system (Agena Bioscience, San Diego, CA, USA). Genotyping of SNPs *CYP2B6* 516G➔T (rs3745274), *CYP2B6* 983T➔C (rs28399499), and *CYP2B6* 15582C➔T (rs4803419) were performed. Missing SNP data were imputed. Genotyping was done at Inqaba Biotechnical Industries, Pretoria, South Africa. Laboratory personnel were blinded to the clinical data. Metaboliser genotype groups for *CYP2B6* were assigned as follows: extensive metaboliser (*CYP2B6* 15882CC-516GG-983TT or *CYP2B6* 15882CT-516GG-983TT), intermediate metabolizer (*CYP2B6* 15882TT-516GG-983TT, *CYP2B6* 15882CC-516GT-983TT, *CYP2B6* 15882CC-516GG-983CT, *CYP2B6* 15882CT-516GT-983TT or *CYP2B6* 15882CT-516GG-983CT), or slow metabolizer (*CYP2B6* 15882CC-516TT-983TT, *CYP2B6* 15882CC-516GT-983CT or *CYP2B6* 15882CC-516GG-983CC) [12].

### Statistical Analyses

Medians (interquartile ranges) and proportions were used to describe continuous and categorical data, respectively. Line graphs and box plots created using R software [15] were used to depict percentage change from baseline to 48 weeks follow-up for weight, central and peripheral fat depots, and lean body mass.

Outcome variables were percentage change from baseline to week 48 of weight, central and peripheral fat depots, and lean body mass. We used Kruskal-Wallis equality-of-populations rank test to assess for between group differences in the outcome variables among the *CYP2B6* metaboliser genotypes in the efavirenz arm; if this was significant (*P* < .050) we performed Dunn pairwise comparison with Bonferroni correction for multiple comparisons. These analyses were also stratified by sex. Furthermore, we compared all outcome variables between the CYP2B6 extensive metabolizers in the efavirenz arm the dolutegravir arm using the 2-sample Wilcoxon rank-sum test.

Univariate and multivariate linear regression models were developed and robust standard errors obtained, to assess percentage weight change from baseline to week 48 among *CYP2B6* metaboliser genotypes and the dolutegravir arm. A priori selected covariates assessed were age; sex; baseline body mass index (BMI), CD4 count, and HIV-1 RNA; and the following self-reported treatment-emergent adverse events: gastrointestinal (nausea/vomiting), neuropsychiatric (anxiety, depression, and psychosis), and insomnia. For the final multivariate model we used the backward elimination method, starting with the full model and removing the most insignificant covariates at each step. We retained age and sex in the final model. All statistical analyses were performed using Stata (version 16.0) [16].

## RESULTS

**Figure 1.**
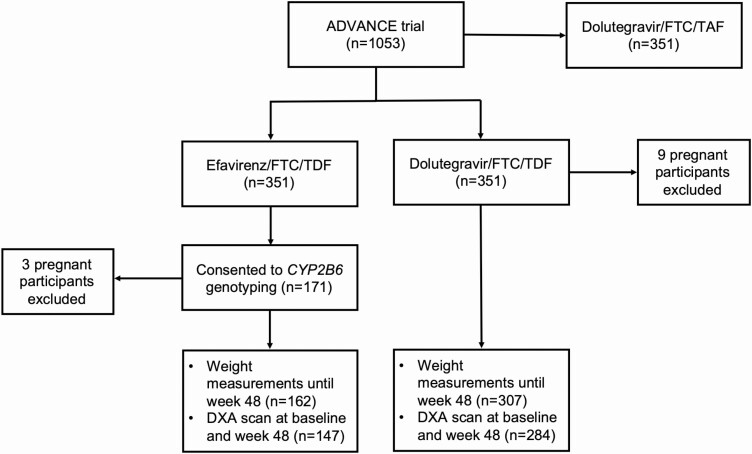
Flow diagram of ADVANCE trial participants enrolled. Abbreviations: DXA, dual-energy x-ray absorptiometry; FTC, emtricitabine; TAF, tenofovir alafenamide; TDF, tenofovir disoproxil fumarate.

Participant flow is shown in Figure 1. In total, 171/351 (48.7%) participants in the efavirenz arm agreed to genetic testing and had *CYP2B6* genotyping performed. Three participants became pregnant during the 48 weeks and were not included in the final analyses. All 3 polymorphisms were successfully genotyped and were in Hardy-Weinberg equilibrium (*P* > .100). None of the polymorphisms were in linkage disequilibrium with each other. The minor allele frequency of *CYP2B6* 516G→T was 0.39, with 67 (39.9%) homozygous for GG, 72 (42.8%) heterozygous for GT, and 29 (17.3%) homozygous for TT. The minor allele frequencies of the 3 *CYP2B6* polymorphisms were: 516G→T 0.39 (67 homozygous for GG, 72 heterozygous for GT, and 29 homozygous for TT); 983T→C 0.10 (137 homozygous for TT, 29 heterozygous for TC, and 2 homozygous for CC); and 15582C→T 0.05 (152 homozygous for CC, 15 heterozygous for TC, and 1 homozygous for TT).

The baseline characteristics of enrolled participants were balanced across all groups (Table 1). The baseline characteristics and weight gain from baseline to week 48 of participants in the efavirenz arm who consented to genetic testing did not differ from those who did not consent to genetic testing (Supplementary Table 1). Self-reported treatment-emergent adverse events were captured at each follow-up visit: nausea and vomiting were more common among the *CYP2B6* slow metabolizers (5/45; 11.1%), and insomnia was more common among the *CYP2B6* extensive metabolizers (5/49; 10.2%) and in the dolutegravir arm (22/342; 6.4%) (Supplementary Table 2).

**Table 1.** Baseline Characteristics of Enrolled Participants

	Efavirenz/FTC/TDF by *CYP2B6* Metabolizer Genotype			Dolutegravir/FTC/TDF (n = 342)
	Extensive (n = 49)	Intermediate (n = 74)	Slow (n = 45)	
**Age (y), median (IQR)**	32 (26–37)	31 (27–36)	33 (29–37)	32 (27–37)
**Sex (women), n (%)**	25 (51.0)	47 (63.5)	22 (48.9)	199 (58.2)
**Race (Black), n (%)**	49 (100)	74 (100)	45 (100)	342 (100)
**BMI (kg/m** ^**2**^ **), median (IQR)**	21.4 (19.7–25.2)	24.4 (21.4–28.3)	23.9 (20.4–27.5)	22.9 (20.1–27.0)
**CD4 count (cells/µL), median (IQR)**	286 (181–381)	303 (176–417)	261 (159–384)	274 (160–424)
**HIV-1 RNA (log**_**10**_ **), median (IQR)**	4.4 (3.9–4.8)	4.3 (3.5–5.1)	4.6 (4.0 to 5.1)	4.4 (3.8–4.9)

Abbreviations: BMI, body mass index; FTC, emtricitabine, HIV-1, human immunodeficiency virus type 1; IQR, interquartile range; TDF, tenofovir disoproxil fumarate.

### Changes in Weight

**Figure 2.**
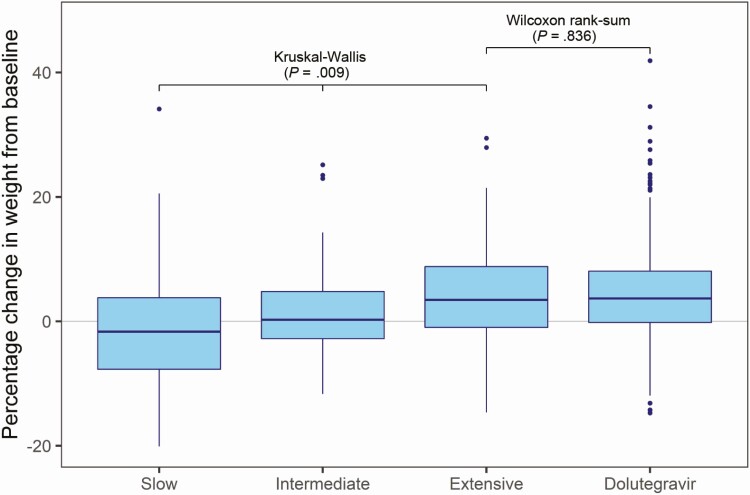
Percentage change (median; IQR) in weight (boxes indicate IQR, horizontal solid line is the median, vertical lines are ranges, solid circles are outliers) from baseline to week 48 by *CYP2B6* metaboliser genotype (extensive, intermediate, and slow) in the efavirenz arm, and the dolutegravir arm. Abbreviation: IQR, interquartile range.

**Figure 3.**
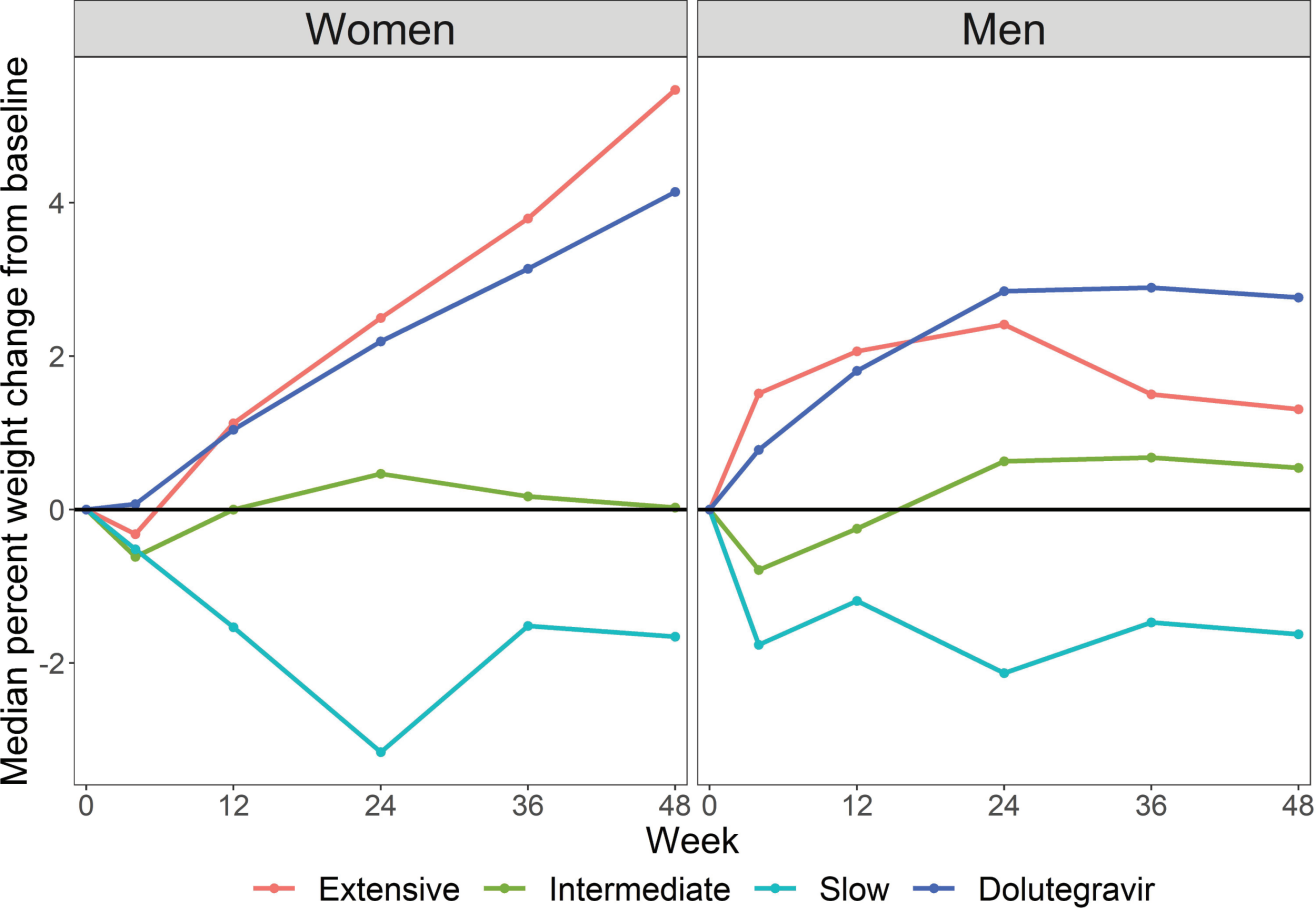
Median percentage weight change from baseline over 48 weeks by *CYP2B6* metaboliser genotype (extensive, intermediate, and slow) in the efavirenz arm and the dolutegravir arm (stratified by sex).

Box plots of percentage change in weight from baseline to week 48 are shown by *CYP2B6* metabolizer genotype in the efavirenz arm and in the dolutegravir arm in Figure 2. In the efavirenz arm percentage weight gain was greatest in *CYP2B6* extensive metabolizers (median 3.5%; interquartile range [IQR] −1.0, 8.9) followed by intermediate metabolizers (median 0.3%; IQR −2.8, 4.8), whereas slow metabolizers lost weight (median −1.7%; IQR −7.7, 4.2) (Kruskal-Wallis *P* = .009) (Supplementary Table 3). There was no difference in weight gain between *CYP2B6* extensive metabolizers in the efavirenz arm and participants in the dolutegravir arm (Wilcoxon rank-sum *P* = .836) (Figure 2). Percentage change in weight from baseline over 48 weeks, stratified by sex, is shown in Figure 3. Weight gain was more marked in women than in men, and weight increased linearly in women but appeared to reach a plateau after 24 weeks in men. *CYP2B6* metabolizer genotype was associated with percentage change in weight from baseline to week 48 in women (Kruskal-Wallis *P* = .010) but not in men (Kruskal-Wallis *P* = .410) (Supplementary Figure 1 and Supplementary Table 4).

Univariate linear regression for the outcome of percentage change in weight from baseline to week 48 is shown in Table 2. Self-reported treatment-emergent adverse events were not associated with weight change from baseline to week 48 and were not included in the multivariate model. On multivariate linear regression the following variables were associated with weight gain: baseline CD4 count, baseline HIV-1 RNA, and *CYP2B6* metaboliser genotype. With the dolutegravir arm as the referent group, *CYP2B6* intermediate and slow metabolizers in the efavirenz arm were strongly associated with weight change (*P* = .002 and *P* = .001, respectively), whereas extensive metabolizers had similar percentage change in weight (*P* = .925) (Table 2). Sex was not independently associated with percentage change in weight.

**Table 2.** Univariate and Multivariate Linear Regression for the Outcome of Percentage Change in Weight from Baseline to Week 48 (n = 469)

Covariate	Univariate Associations		Multivariate Associations	
	Estimate (95% CI)	*P*-value	Estimate (95% CI)	*P*-value
**Age (per 5 y increase)**	−.014 (−.537 to .509)	.958	−.038 (−.539 to .463)	.881
**Sex**				
Women	Referent group			
Men	−.618 (−2.127 to .890)	.421	−1.318 (−2.793 to .156)	.080
**Baseline BMI (per 1 kg/m** ^**2**^ **increase)**	−.119 (−.244 to .006)	.061		
**Baseline CD4 count (per 50 cells/µL increase)**	−.399 (−.567 to −.232)	<.001	−.253 (−.417 to −.089)	.003
**Baseline HIV-1 RNA (per 1 log**_**10**_ **increase)**	2.569 (1.651 to 3.487)	<.001	2.122 (1.169 to 3.074)	<.001
**HIV-1 RNA suppression (<50 copies/mL) at week 48**				
No	Referent group			
Yes	.949 (−1.591 to 2.561)	.377		
**ARV category**				
**Dolutegravir/FTC/TDF arm**	Referent group			
**Efavirenz/FTC/TDF arm**				
Extensive	.042 (−2.726 to 2.642)	.976	−.119 (−2.607 to 2.369)	.925
Intermediate	−2.889 (−4.774 to −1.005)	.003	−2.917 (−4.763 to −1.071)	.002
Slow	−4.884 (−7.993 to −1.776)	.002	−5.304 (−8.352 to −2.256)	.001
**Treatment emergent gastrointestinal adverse events** ^a^				
No	Referent group			
Yes	.661 (−2.591 to 3.913)	.690		
**Treatment emergent neuropsychiatric adverse events** ^b^				
No	Referent group			
Yes	3.810 (−5.091 to 12.711)	.401		
**Treatment emergent insomnia**				
No	Referent group			
Yes	.780 (−3214 to 4.774)	.701		

Abbreviations: ARV, antiretroviral, BMI, body mass index, CI, confidence interval; FTC, emtricitabine, HIV-1, human immunodeficiency virus type 1; TDF, tenofovir disoproxil fumarate.

^a^Nausea and vomiting.

^b^Anxiety, depression, or psychosis.

### Changes in Fat and Lean Body Mass Distribution on DXA

There were major sex differences in changes of regional fat and lean body mass distribution on DXA, and all analyses below are stratified by sex; analyses of participants not stratified by sex are shown in the supplementary material (Supplementary Figures 2 and 3 and Supplementary Table 3).

**Figure 4.**
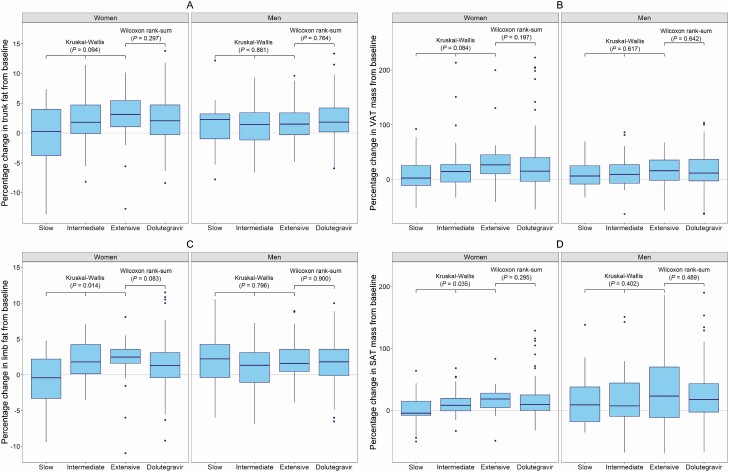
Percentage change (median; IQR) in trunk fat (*A*), VAT (*B*), limb fat (*C*), and SAT (*D*) stratified by sex (boxes indicate IQR, horizontal solid line is the median, vertical lines are ranges, solid circles are outliers) from baseline to week 48 by *CYP2B6* metaboliser genotype (extensive, intermediate, and slow) in the efavirenz arm, and the dolutegravir arm. Abbreviations: IQR, interquartile range; SAT, subcutaneous adipose tissue; VAT, abdominal visceral adipose tissue.

Percentage change in trunk fat and VAT mass from baseline to week 48 was not associated with *CYP2B6* metabolizer genotype in the efavirenz arm in women (Kruskal-Wallis *P* = .094 and *P* = .084, respectively) nor in men (Kruskal-Wallis *P* = .881 and *P* = .617, respectively) (Figure 4). Percentage change in both trunk fat and VAT was similar between *CYP2B6* extensive metabolizers and the dolutegravir arm in both sexes (Figure 4).

Percentage change in limb fat and SAT mass from baseline to week 48 was associated with *CYP2B6* metaboliser genotype in the efavirenz arm in women (Kruskal-Wallis *P* = .014 and *P* = .035, respectively) (Supplementary Tables 4) but not in men (Kruskal-Wallis *P* = .796 and *P* = .402, respectively) (Figure 4). Percentage change in both limb fat and SAT was similar between *CYP2B6* extensive metabolizers and the dolutegravir arm in both sexes (Figure 4).

Percentage change in lean body mass from baseline to week 48 was associated with *CYP2B6* metabolizer genotype among women in the efavirenz arm (Kruskal-Wallis *P* = .023) (Supplementary Table 4) but not among men (Kruskal-Wallis *P* = .850) (Supplementary Figure 4). Percentage change in lean body mass was similar between *CYP2B6* extensive metabolizers and the dolutegravir arm in both sexes (Supplementary Figure 4).

## DISCUSSION

We found that *CYP2B6* metaboliser genotype was strongly associated with weight gain over 48 weeks in the efavirenz arm: extensive metabolizers gained the most weight, whereas slow metabolizers lost weight. *CYP2B6* extensive metabolizers in the efavirenz arm had similar weight gain to participants in the dolutegravir arm. These findings suggest that the greater weight gain reported on dolutegravir-based ART compared with efavirenz-based ART could be due to impaired weight gain in PLWH with *CYP2B6* intermediate or slow metaboliser genotypes, who likely had increased efavirenz concentrations causing toxicity, rather than off target effects of dolutegravir affecting appetite or metabolism. There were striking sex differences in weight changes on ART: women gained more weight than men and *CYP2B6* metabolizer genotype was associated with central and peripheral fat changes on DXA over 48 weeks in women but not in men.

Our finding that weight gain was similar in *CYP2B6* extensive metabolizers in the efavirenz arm and the dolutegravir arm, with *CYP2B6* intermediate and slow metabolizers gaining less weight, are consistent with an observational study of PLWH on efavirenz-based ART switching to InSTI-based ART: *CYP2B6* slow and intermediate metabolizers gained more weight than *CYP2B6* extensive metabolizers after switching [13]. The large weight gain differences observed between dolutegravir-based ART and efavirenz-based ART observed in the NAMSAL and ADVANCE studies could be explained by the higher prevalence of *CYP2B6* slow metabolizers in people of African ancestry [17]. Our finding that baseline CD4 count and HIV-1 RNA were strongly associated with weight gain is in keeping with findings from a pooled analysis of 8 trials in ART-naive participants [1]. Our finding that women gained more weight on ART than men is well established [1, 18]. We also found that the trajectories of weight gain differed by sex, with women gaining weight linearly and men reaching a plateau, which is consistent with an observational cohort study showing that women gained weight linearly over 3.5 years whereas men gained weight linearly over 6–12 months, followed by more gradual weight gain [18].

Rates of virologic suppression were similar in all groups in our study (Supplementary Figure 5), so the weight differences we observed were not due to ART failure. We explored the possibility that impaired weight gain in participants in the efavirenz arm with loss-of-function polymorphisms in *CYP2B6* could be explained by an increased incidence of self-reported treatment-emergent gastrointestinal or neuropsychiatric adverse events due to high efavirenz concentrations. Although we found a higher proportion of self-reported nausea and vomiting among *CYP2B6* slow metabolizers, none of the selected treatment-emergent adverse events were significantly associated with weight change at week 48 on univariate linear regression.

The association between *CYP2B6* metaboliser genotype and fat distribution in women was more marked peripherally (SAT and limb fat) than centrally (VAT and trunk fat). We hypothesize that these differences in regional fat deposition could be due to the mitochondrial toxicity and impaired adipocyte differentiation of efavirenz, both of which are concentration dependent [10, 19]. Efavirenz has deleterious effects on adipogenesis (inhibiting the expression of genes controlling adipogenesis and lipid accretion and reducing the release of adipokines) and increases release of pro-inflammatory cytokines, which are catabolic [10, 20]. Our observation that changes in regional fat by *CYP2B6* metaboliser genotype in the efavirenz arm was limited to women could be contributed to by higher efavirenz plasma concentrations in women [21, 22]; larger studies with longer follow-up may show similar findings in men.

Our study had limitations. First, only about half the participants in the efavirenz arm of ADVANCE agreed to genotyping. However, their baseline characteristics were similar to participants who did not agree to genotyping. Second, ours is a post hoc analysis of the ADVANCE study, with no formal sample size calculation to ensure adequate power, which increases the risk of chance findings. Third, our findings were in an African population of ART-naive participants and may not be generalizable to other populations. Fourth, we only had weight data over 48 weeks; longer term data will be important to determine the consequences of weight gain and weight trajectories over a longer period. Fifth, we did not perform *CYP2B6* genotyping on participants in the dolutegravir arm to explore potential associations with weight gain. Finally, isoniazid was prescribed as tuberculosis preventive therapy for most participants in ADVANCE for the first 48 weeks. Isoniazid has an inhibitory effect on the metabolism of efavirenz among *CYP2B6* slow metabolizers, resulting in about a 50% increase in efavirenz exposure [23, 24], which would accentuate concentration dependent toxicity. Strengths of our study include the randomized trial design of ADVANCE, the use of the same dual nucleoside/nucleotide reverse transcriptase inhibitors in both arms, and the use of DXA to evaluate longitudinal changes in regional fat.

In conclusion, *CYP2B6* metaboliser genotype was associated with weight gain among all participants starting efavirenz-based ART and with fat distribution in women but not in men. Our finding that weight gain was similar in CYP2B6 extensive metabolizers in the efavirenz arm and in the dolutegravir arm, together with the finding by Sax et al [1] that newer and safer antiretrovirals are associated with more weight gain than older antiretrovirals, suggests that weight gain on newer ART regimens is a return to health phenomenon rather than off target effects of newer antiretrovirals from different classes on appetite or metabolism.

## Supplementary Data

Supplementary materials are available at *Clinical Infectious Diseases* online. Consisting of data provided by the authors to benefit the reader, the posted materials are not copyedited and are the sole responsibility of the authors, so questions or comments should be addressed to the corresponding author.

ciaa1073_suppl_Supplemental_Figure_S1

ciaa1073_suppl_Supplemental_Figure_S2

ciaa1073_suppl_Supplemental_Figure_S3

ciaa1073_suppl_Supplemental_Figure_S4

ciaa1073_suppl_Supplemental_Figure_S5

ciaa1073_suppl_Supplemental_Material

## Notes

***Acknowledgments.*** DXA measurements, including estimated VAT and SAT, were conducted by Shane Norris, Developmental Pathways for Health Research Unit, Faculty of Health Sciences, University of the Witwatersrand, Johannesburg, South Africa. DNA extraction from whole blood samples was done at the Sydney Brenner Institute for Molecular Bioscience (SBIMB) Biobank, Johannesburg, South Africa.

***Financial support.*** This work was supported by the National Research Foundation of South Africa (119078), Wellcome Trust through an investigator award (212265/Z/18/Z) and core funding form the Wellcome Centre for Infectious Diseases Research in Africa (203135/Z/16/Z), as well as the South African Medical Research Council under a self-initiated research grant to P. S. (the views and opinions expressed are those of the authors and do not necessarily represent the official views of the SA MRC).

***Potential conflicts of interest.*** G. M. reports advisory board fees from ViiV Healthcare, outside the submitted work. M. M. reports speaker fees and honoraria from Gilead Sciences, AbbVie, Cipla, Johnson and Johnson, Sanofi, Pfizer, ViiV Healthcare, Mylan and Southern African HIV Clinicians Society; and conference/meeting sponsorship from Johnson and Johnson, BD, Gilead, Merck, Cipla, Mylan and Canopy Growth, outside the submitted work. F. V. reports receiving drug donations from ViiV Healthcare and Gilead Sciences for investigator-led clinical studies, and honoraria for talks and board membership for: Gilead, ViiV, Mylan, Merk, Adcock-Ingram, Aspen, Abbott, Roche, J&J, outside the submitted work. All other authors report no potential conflicts. All authors have submitted the ICMJE Form for Disclosure of Potential Conflicts of Interest. Conflicts that the editors consider relevant to the content of the manuscript have been disclosed.
