# Supplementary figures and images for: CYP2B6 Genotype and Weight Gain Differences Between Dolutegravir and Efavirenz
Source: Clin Infect Dis. 2020 Sep 22;73(11):e3902–9. doi: 10.1093/cid/ciaa1073 (PMC8653639; doi:10.1093/cid/ciaa1073)

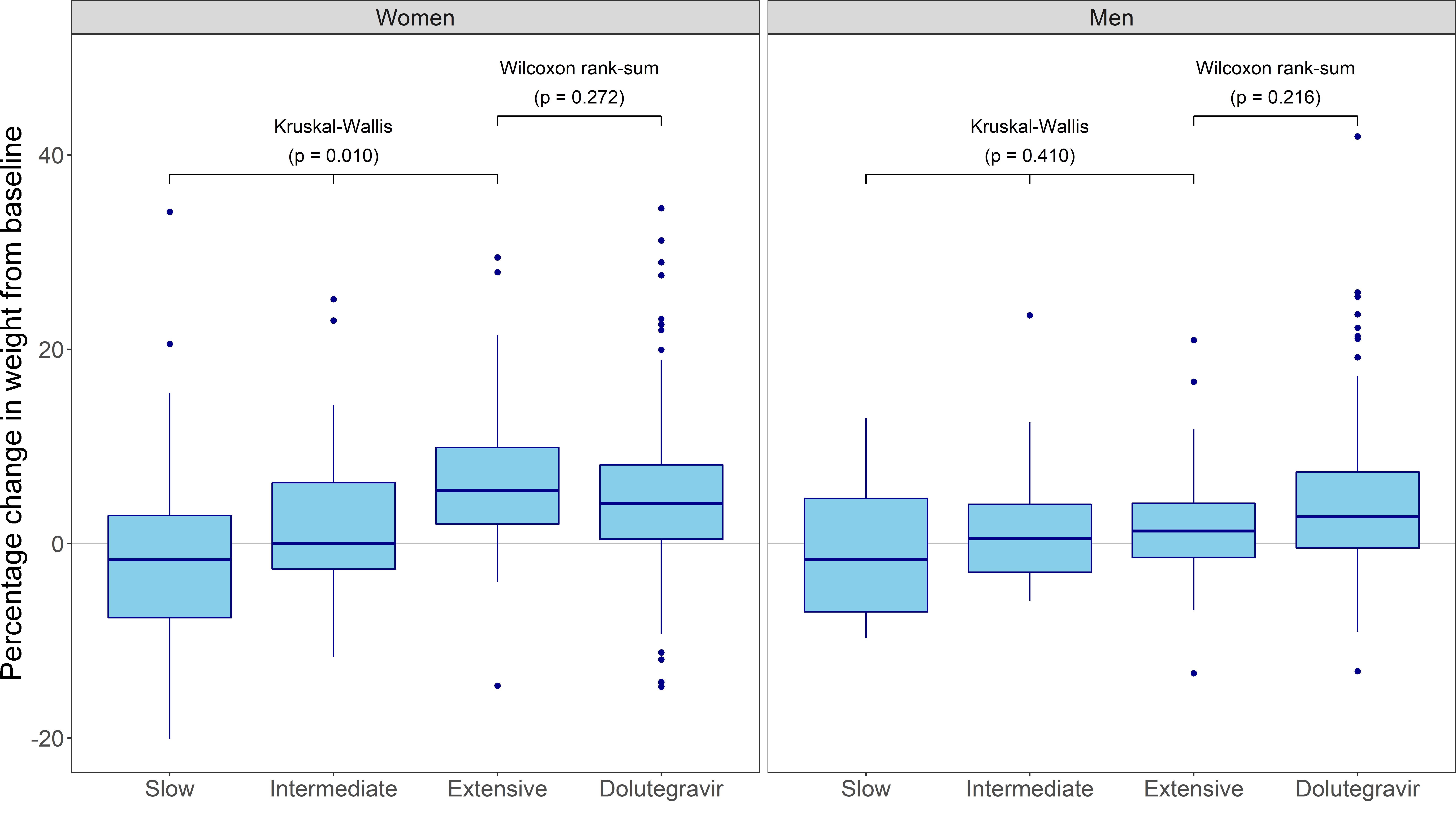

Supplement: ciaa1073_suppl_Supplemental_Figure_S1 [file ciaa1073_suppl_supplemental_figure_s1.jpeg]

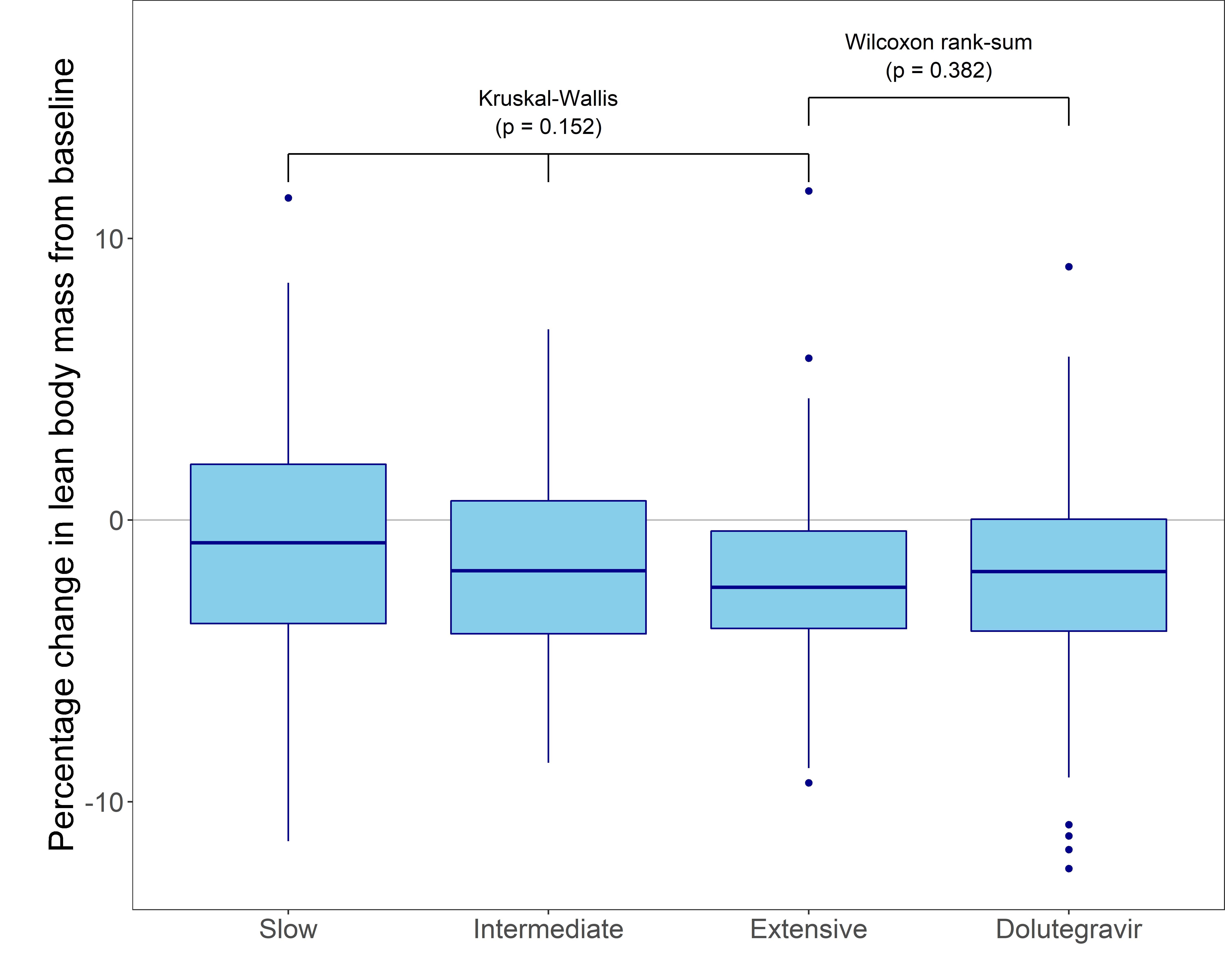

Supplement: ciaa1073_suppl_Supplemental_Figure_S3 [file ciaa1073_suppl_supplemental_figure_s3.jpeg]

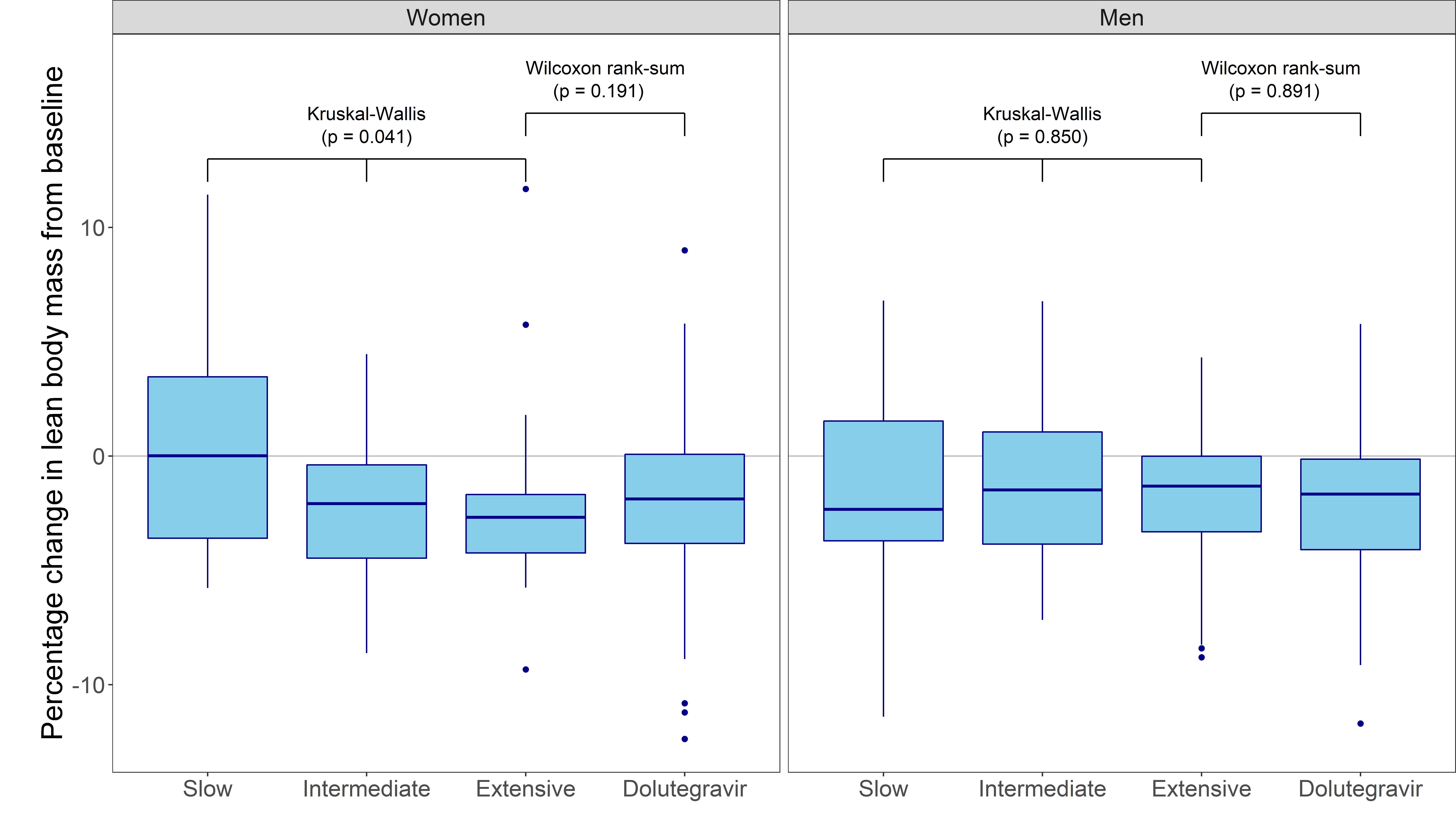

Supplement: ciaa1073_suppl_Supplemental_Figure_S4 [file ciaa1073_suppl_supplemental_figure_s4.jpeg]

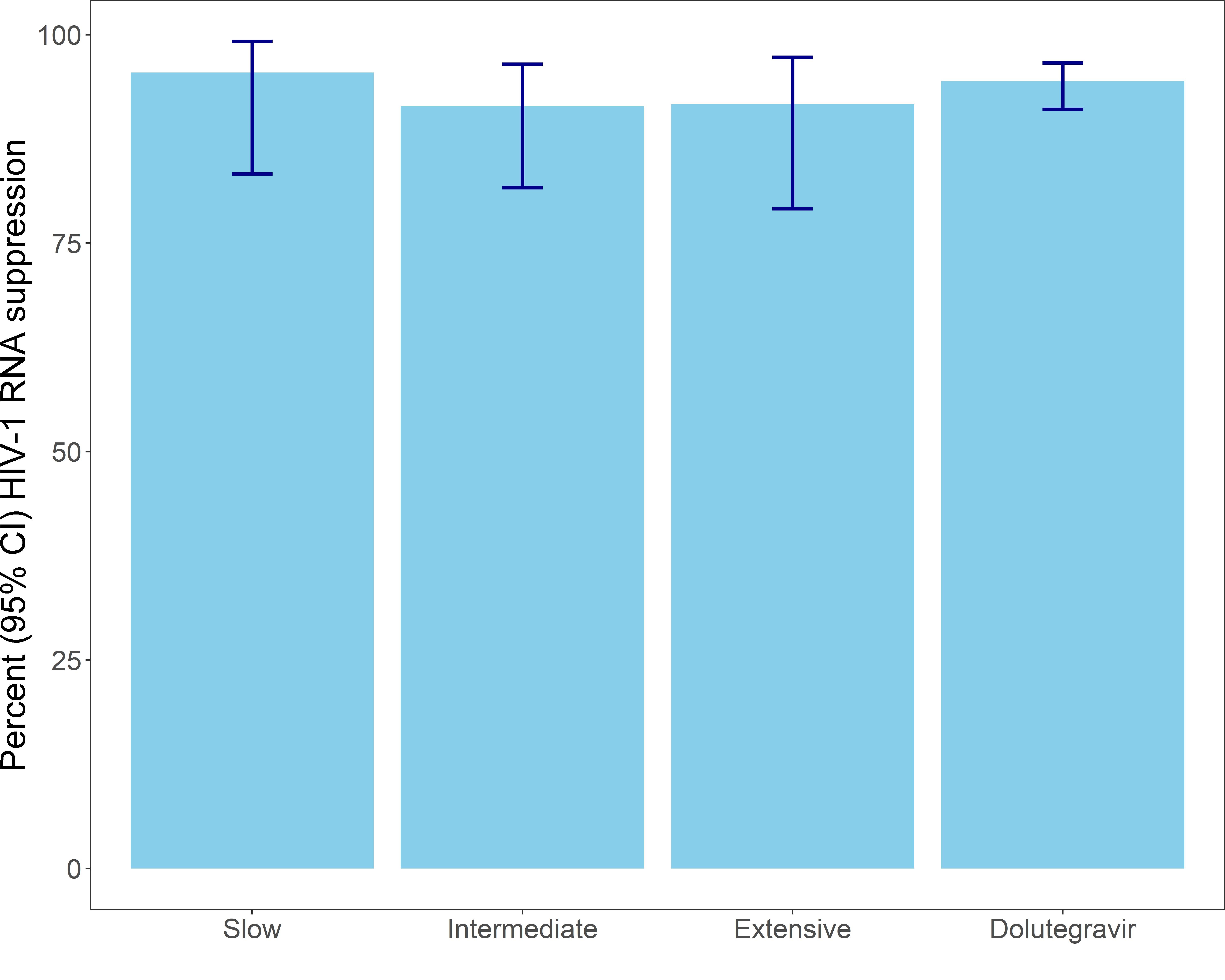

Supplement: ciaa1073_suppl_Supplemental_Figure_S5 [file ciaa1073_suppl_supplemental_figure_s5.jpeg]
