# Supplementary material for: CYP2B6 Genotype and Weight Gain Differences Between Dolutegravir and Efavirenz
Source: Clin Infect Dis. 2020 Sep 22;73(11):e3902–9. doi: 10.1093/cid/ciaa1073 (PMC8653639; doi:10.1093/cid/ciaa1073)
Supplement: ciaa1073_suppl_Supplemental_Material [file ciaa1073_suppl_supplemental_material.docx]

**Supplemental Table 1:** Comparison of baseline characteristics between participants in the efavirenz arm that consented to genetic testing versus participants who did not consent to genetic testing (pregnant cases were excluded from this comparison) (two-sample Wilcoxon rank-sum test for continuous variables and Fisher's exact for categorical variables)

|  | **Participants consented to genetic testing in the Efavirenz/FTC/TDF arm**  **(n=168)** | **Participants not consented to genetic testing in the Efavirenz/FTC/TDF arm (n=178)** | **p-value** |
| --- | --- | --- | --- |
| **Age (years), median (IQR)** | 32 (28 to 37) | 31 (27 to 38) | 0.827 |
| **Sex (women), n (%)** | 94 (56.0) | 102 (57.3) | 0.829 |
| **Race (black), n (%)** | 168 (100) | 178 (100) | 1.000 |
| **BMI (kg/m^2^), median (IQR)** | 23.7 (20.2 to 27.5) | 22.4 (19.9 to 25.6) | 0.060 |
| **CD4 count (cells/µL), median (IQR)** | 291 (172 to 406) | 307 (182 to 488) | 0.282 |
| **HIV-1 RNA (log_10_), median (IQR)** | 4.4 (3.7 to 5.0) | 4.3 (3.7 to 4.9) | 0.296 |
| **Percentage weight gain from baseline to week 48, median (IQR)** | 0.6 (-2.9 to 6.6) | 0.6 (-3.7 to 7.7) | 0.887 |

FTC = Emtricitabine, TDF = Tenofovir disoproxil fumarate, BMI = Body mass index

**Supplemental Table 2:** Summary of self-reported treatment emergent adverse events from baseline to week 48 by *CYP2B6* metaboliser genotype in the efavirenz arm and the dolutegravir arm

|  | **Efavirenz/FTC/TDF by *CYP2B6* metaboliser genotype** | | | **Dolutegravir/FTC/TDF (n=342)** |
| --- | --- | --- | --- | --- |
|  | **Extensive**  **(n=49)** | **Intermediate**  **(n=74)** | **Slow**  **(n=45)** |  |
| **Gastrointestinal treatment emergent adverse events** |  |  |  |  |
| **Nausea/Vomiting, n (%)** | 3 (6.1) | 4 (5.4) | 5 (11.1) | 16 (4.7) |
| **Neuropsychiatric treatment emergent adverse events** |  |  |  |  |
| **Insomnia, n (%)** | 5 (10.2) | 2 (2.7) | 2 (4.4) | 22 (6.4) |
| **Psychosis, n (%)** | 0 | 0 | 0 | 1 (0.3) |
| **Anxiety, n (%)** | 0 | 0 | 0 | 2 (0.6) |
| **Depression, n (%)** | 1 (2) | 1 (1.4) | 2 (4.4) | 6 (1.8) |

FTC = Emtricitabine, TDF = Tenofovir disoproxil fumarate

**Supplemental Table 3:** Dunn’s p-values for pairwise comparison by outcome among the *CYP2B6* metaboliser genotypes in the efavirenz arm (Only performed where the Kruskal-Wallis equality-of-populations rank test for between group differences was significant (p<0.050))

| **Outcome** |  | **Intermediate** | **Slow** |
| --- | --- | --- | --- |
| Percentage weight change from baseline to week 48 | **Extensive** | 0.068 | 0.004 |
|  | **Intermediate** | - | 0.267 |
| Percentage SAT change from baseline to week 48 | **Extensive** | 0.111 | 0.024 |
|  | **Intermediate** | - | 0.560 |

SAT = subcutaneous adipose tissue

**Supplemental Table 4:** Dunn’s p-values for pairwise comparison by outcome among the *CYP2B6* metaboliser genotypes in the efavirenz arm in women (Only performed where the Kruskal-Wallis equality-of-populations rank test for between group differences was significant (p<0.050))

| **Outcome** |  | **Intermediate** | **Slow** |
| --- | --- | --- | --- |
| Percentage weight change from baseline to week 48 | **Extensive** | 0.028 | 0.006 |
|  | **Intermediate** | - | 0.454 |
| Percentage limb fat change from baseline to week 48 | **Extensive** | 1.000 | 0.014 |
|  | **Intermediate** | - | 0.015 |
| Percentage SAT change from baseline to week 48 | **Extensive** | 0.284 | 0.015 |
|  | **Intermediate** | - | 0.136 |
| Percentage lean body mass change from baseline to week 48 | **Extensive** | 0.826 | 0.027 |
|  | **Intermediate** | - | 0.054 |

SAT = subcutaneous adipose tissue

**Supplemental Figure 1:** Percentage (median; IQR) change in weight stratified by sex (boxes indicate interquartile range, horizontal solid line is the median, vertical lines are ranges, solid circles are outliers) from baseline to week 48 by *CYP2B6* metaboliser genotype in the efavirenz arm, and the dolutegravir arm

**Supplemental Figure 2:** Percentage change (median; IQR) in trunk fat (A), VAT (B), limb fat (C), and SAT (D) (boxes indicate interquartile range, horizontal solid line is the median, vertical lines are ranges, solid circles are outliers) from baseline to week 48 by *CYP2B6* metaboliser genotype in the efavirenz arm, and the dolutegravir arm (VAT = abdominal visceral adipose tissue, SAT = subcutaneous adipose tissue)

**Supplemental Figure 3:** Percentage change (median; IQR) in lean body mass (boxes indicate interquartile range, horizontal solid line is the median, vertical lines are ranges, solid circles are outliers) from baseline to week 48 by *CYP2B6* metaboliser genotype in the efavirenz arm, and the dolutegravir arm

**Supplemental Figure 4:** Percentage change (median; IQR) in lean body mass stratified by sex (boxes indicate interquartile range, horizontal solid line is the median, vertical lines are ranges, solid circles are outliers) from baseline to week 48 by *CYP2B6* metaboliser genotype in the efavirenz arm, and the dolutegravir arm

**Supplemental Figure 5:** Virological suppression (HIV-1 RNA <50 copies/mL) rate at week 48 by *CYP2B6* metaboliser genotype in the efavirenz arm, and the dolutegravir arm (Fisher’s exact test for between group comparisons, p=0.638)
